# Implications for changes in *Anophelesdarlingi* biting behaviour in three communities in the peri-Iquitos region of Amazonian Peru

**DOI:** 10.1186/s12936-015-0804-2

**Published:** 2015-07-30

**Authors:** Marta Moreno, Marlon P Saavedra, Sara A Bickersmith, William Lainhart, Carlos Tong, Freddy Alava, Joseph M Vinetz, Jan E Conn

**Affiliations:** Division of Infectious Diseases, Department of Medicine, University of California San Diego, La Jolla, CA USA; Asociación Benéfica PRISMA, Lima, Peru; Wadsworth Center, New York State Department of Health, Albany, NY USA; Department of Biomedical Sciences, School of Public Health, University at Albany (State University of New York), Albany, NY USA; Instituto de Medicine Tropical “Alexander von Humboldt”, Universidad Peruana Cayetano Heredia, Lima, Peru; Ministry of Health, Iquitos, Peru

**Keywords:** Malaria, *Anopheles darlingi*, Entomological inoculation rate, Human landing catch, Amazonian Peru

## Abstract

**Background:**

Malaria transmission in the peri-Iquitos region of Amazonian Peru has been designated as seasonal and hypo-endemic with recently described hyper-endemic hotspots. Despite relatively recent distribution of long-lasting insecticidal bed nets (LLINs), malaria in Amazonian Peru persists and increased substantially in 2014 compared to previous years. *Anopheles darlingi*, identified as the main malaria vector, is known for its variable behaviour depending on locality and environment.

**Methods:**

To evaluate vector biology metrics in relation to seasonality and malaria transmission, mosquito collections were carried out in three localities in the peri-Iquitos region, Loreto, Peru in 2011–2012. Human landing catch (HLC) collection method, Shannon (SHA) and CDC trap types were compared for effectiveness in a neotropical setting. Abundance, human biting rate and entomological inoculation rate (EIR) were measured to provide an updated view of transmission patterns post-LLIN distribution.

**Results:**

HLC collected significantly more anopheline mosquitoes than SHA and CDC light traps. *Anopheles darlingi* was the most prevalent species in all three villages (84% overall). Biting patterns varied depending on trap type, season and village. EIR varied temporally (monthly) and spatially and the highest (2.52) occurred during the 2012 malaria outbreak in Cahuide. Unexpectedly there was a high infection rate (1.47 and 1.75) outside the normal malaria transmission season, coincident with a second local outbreak in Cahuide. The first identification of *Anopheles dunhami* and *Anopheles oswaldoi* C in Peru, using molecular markers, is also reported in this study.

**Conclusion:**

These data underscore the importance of HLC as the most meaningful collection method for measuring vector biology indices in this region. The highest monthly EIR provides additional evidence of seasonal transmission in riverine localities correlated with high river levels, and *An. darlingi* as the only contributor to transmission. The trend of an increase in outdoor-biting together with early-evening infected mosquitoes may undermine the effectiveness of LLINs as a primary malaria intervention.

## Background

Dynamics, behaviour and host preference of anopheline species significantly affect malaria transmission in endemic areas. To elucidate the unique factors associated with maintenance in low or seasonal malaria transmission regions, such as the Amazon, entomological longitudinal studies that take into account seasonal and between-year variations are particularly informative for predictive models [1, 2]. The Peruvian Amazon has been defined as a low transmission area [3], with marked seasonal transmission, i.e., increased case numbers during the rainy season, from January to June, and low numbers during the dry season. However, newly described hyper-endemic foci linked to occupational activities [4] suggest considerable spatial variation in endemicity levels with important implications for epidemiology and efforts to reduce human-vector contact. During the past 4 years (from 11,779 in 2011 to 60,186 in 2014) there has been an annual increase in malaria cases in Loreto Department, Amazonian Peru. The proportion of malaria cases in Loreto is 93.69% of the whole country, and the annual parasite index is 58.49 [5, 6].

*Anopheles darlingi* is the main vector in the region although other species, such as *Anopheles benarrochi* s.l., may be involved in local transmission [7, 8]. In the Iquitos area, Andean snowmelt combined with rainfall increases river levels (up to 10 m), resulting in large seasonal fluctuations in anopheline abundance [9]. Taken together, these factors directly and indirectly affect malaria dynamics. Furthermore, in Amazonian Brazil, distinctive *An. darlingi* sub-populations have been shown to be adapted to distinctive rainfall regimes, likely promoting year-long transmission [10].

*Anopheles darlingi*’s behaviour is extremely ‘plastic’ (exophagic/endophagic, exophilic/endophilic, opportunistic and highly anthropophilic), and it is difficult to extrapolate from one local epidemiological situation to another [9, 11, 12]. In Brazil, there is evidence of behavioural modification from endophily to exophily as a result of indoor insecticide spraying [13]. Peak biting activity varies depending on environmental variables, insecticide-treated nets (ITN) usage, and sociodemographic characteristics of the human population [14, 15]. Uni-, bi- and trimodal peaks have all been documented from different regions within the range of this species [12].

Entomological inoculation rate (EIR) is used to estimate intensities of malaria transmission and to evaluate the effectiveness of intervention strategies [16, 17]. However, comparisons of this metric across settings can be complicated by differences in sensitivity of sporozoite detection, time-scale and/or collection method. Direct dissection of salivary glands, ELISA assays and molecular techniques to detect *Plasmodium* DNA in the mosquito are the three most commonly used methods [18–20]. To date, infectivity rates of anophelines in the Peruvian Amazon have been based on ELISA [4, 9], and this technique could underestimate EIR because of decreased sensitivity compared to other molecular detection methods, such as PCR [21].

Considering the moderate to high levels of phenotypic and genetic variation in Amazonian *An. darlingi* populations [22–24], but see [25], this study hypothesizes that there will be high seasonal changes in human biting rate (HBR) and EIR within and among three malaria-endemic villages in the Iquitos area. Furthermore, because of the previously documented anthropophilic behaviour of *An. darlingi* [9, 26] significantly more anophelines may be collected using human landing catch (HLC) than with other collection methods. This work can contribute to a better understanding of *An. darlingi* changes in behaviour vis-à-vis malaria transmission in response to vector-based interventions such as long-lasting insecticide nets (LLINs).

## Methods

### Collection sites

**Figure 1:**
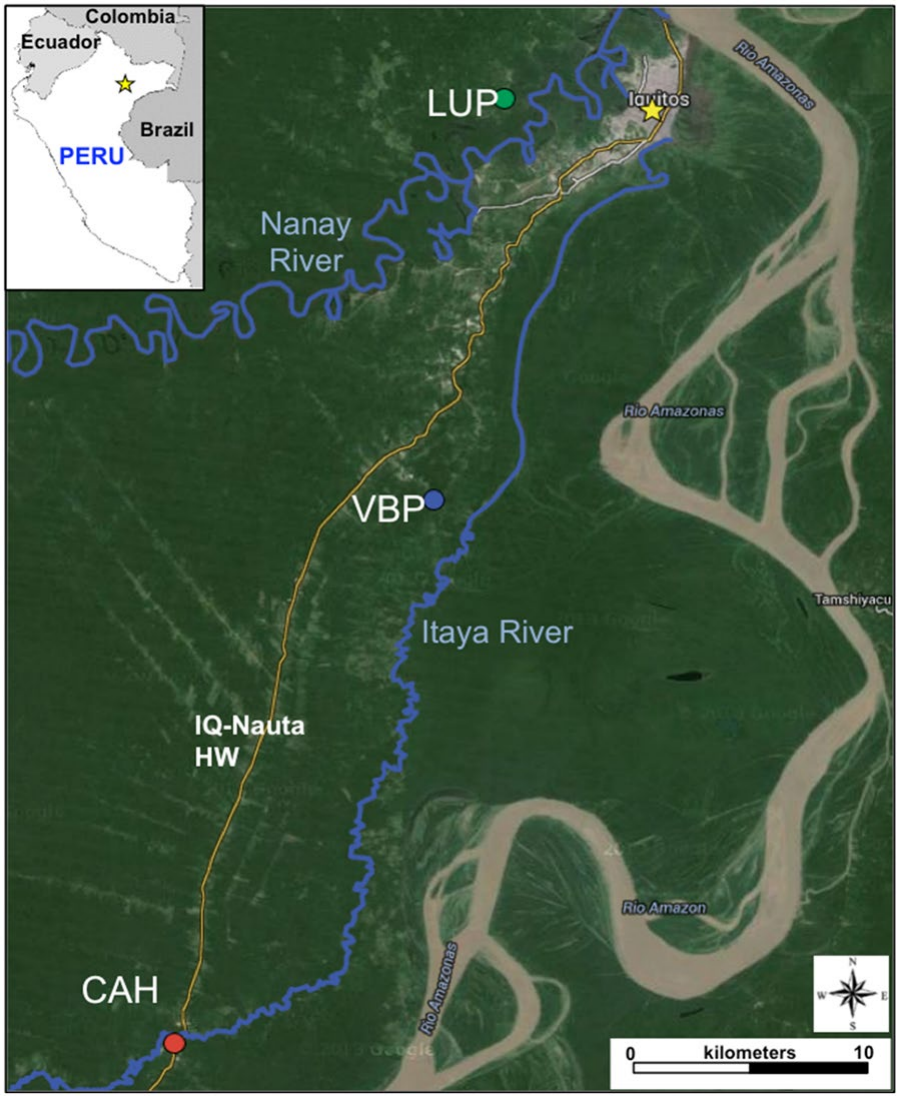
Mosquito collection site in the Iquitos area. Lupuna (LUP) is located on the Nanay River; Villa del Buen Pastor (VBP) and Cahuide (CAH) are on the Itaya River. Both rivers are tributaries of the Amazon. Iquitos city is denoted by a *yellow star.*

A longitudinal study was designed to collect mosquitoes from three localities in the Iquitos area, Loreto Department, Peru during 2011–2012 (Figure 1). San José de Lupuna community (LUP) is a network of four villages located on the Nanay River, a tributary of the Amazon River, and the main occupation of the villagers includes agricultural activities such as mandioca cultivation and charcoal production. Villa del Buen Pastor (VBP) is on the Iquitos-Nauta road, 21 km south of Iquitos. Here, most inhabitants are involved in mixed crop farming and/or fishing. Cahuide (CAH) is a centre of palm roof production and is located where the Iquitos-Nauta road and the Itaya River intersect. Both *Plasmodium vivax* and *Plasmodium falciparum* cases are reported annually for all three villages. At the time of the field collections, the only major intervention in the localities was the use of LLINs distributed during 2008–2010 by the PAMAFRO initiative [27]. In 2012, after a local malaria outbreak, the Ministry of Health distributed new bed nets, and currently the inhabitants use either *tocuyos* (locally made cotton nets without insecticide) or LLINs (Table 1). Levels of the Nanay and Itaya rivers from 2011 to 2012 were obtained from Servicio Nacional de Meteorología e Hidrología del Perú [28].

**Table 1:** Bed net coverage in Cahuide (CAH), Lupuna (LUP) and Villa Buen Pastor (VBP) in 2012

Locality	House with bed net^a^ (%)	House with LLIN (%)	No. houses
CAH	276 (99.6)	125 (45.1)	277
LUP	211 (100)	185 (87.7)	211
VBP	–	56 (100)^b^	63^c^

^a^Bed net includes LLIN and non-impregnated local bed nets.

^b^Number of houses and LLIN distribution in 2010.

^c^Number of houses in 2013.

### Collection methods

Adult mosquitoes were collected during forty-eight nights in LUP, twenty-four in VBP and sixteen in CAH (four nights/month, every other month), including wet and dry season. HLC and Shannon trap (SHA) collections were performed outdoor for 12 h (18:00–06:00) stationed ~10 m from the house with personnel rotating every 2 h, to account for variation in individual attractiveness to mosquitoes [29]. CDC light traps were used for indoor collection and positioned next to the bed where a person slept under a LLIN. In addition, HLC indoor/outdoor (18:00–22:00) was performed in LUP and CAH in 2012. HBR was calculated with the data obtained from HLC in the 12 h collection. Collected mosquitoes were separated by hour and then morphologically identified by trained personnel using the available keys [30–32]. Specimens were then individually stored in silica gel until DNA extraction.

### Laboratory processing

#### Circumsporozoite protein ELISA assays

The head/thorax from specimens collected in LUP and CAH from 2012 were analysed in pools of five individuals (same species and same collection date/time). The ELISA protocol was performed following Wirtz et al. [18] and can distinguish among circumsporozoite protein (CSP) of *P. falciparum* and *P. vivax* (Pv210 and Pv247). *Plasmodium falciparum* sporozoite ELISA kit MRA-890 and *P. vivax* sporozoite ELISA kit MRA-1028K (deposited by R A Wirtz) were obtained through the Malaria Research and Reference Reagent Resource Center (MR4) as part of the BEI Resources Repository, NIAID, NIH and laboratory-reared female *An. darlingi* were used as negative controls. Optical density was measured at 410 nm in a Bio-Rad ELISA plate reader 30 and 60 min after adding the substrate. The cut-off for positivity was determined by the mean OD value of negative controls for each plate.

#### DNA extraction and PCR assays

Total genomic DNA was extracted from the head/thorax of each specimen from LUP and VBP collected in 2011 using the DNeasy tissue kit (Qiagen, CA, USA). A sub-sample of specimens that could not be identified morphologically (cryptic *Nyssorhynchus* species, damaged specimens, etc.) were identified using ITS2 sequence [33], ITS-RFLP [34] and the BOLD region of *COI* [35]. *Plasmodium* detection in mosquitoes was performed following the PCR–RFLP protocol described in Hasan et al. [20] based on the *PlasmodiumCyt*-*b* gene. Pools of five mosquitoes (same species and same date/time of capture) were tested together; individuals of each positive pool were tested individually. For both detection protocols, sporozoite rates were calculated using the number of positive mosquitoes for *Plasmodium* divided by the number of tested mosquitoes. Monthly EIR was calculated by multiplying the HBR by proportion of infected specimens per month.

### Ethical issues

This study was approved by the Human Subjects Protection Program of the University of California San Diego, La Jolla, and by the Comité de Ética of the Universidad Peruana Cayetano Heredia and Asociación Benéfica PRISMA, Lima, Peru.

## Results

A total of 14,001 female anophelines, 7,066 in LUP, 846 in VBP and 2,447 in CAH, were collected in 48 sampling nights in LUP and VBP and 16 nights in CAH (summarized in Table 2). *Anopheles darlingi* was by far the most abundant anopheline in all three localities in each collection with a contribution by trap type varying in LUP from the lowest 33% in December to the highest 96.4% in June, and 88.9% in February to 100% in June in VBP and in nearly every collection in CAH. In LUP, *Anopheles nuneztovari* s.l. was collected during the most months of the year with up to 31.7% contribution to total mosquito abundance in February. *Anopheles triannulatus* s.l., *Anopheles oswaldoi* s.l. and *An. benarrochi* B were present in lower frequency and were collected primarily in February and April. In contrast, species composition was lower in VBP; *An. darlingi* was the most abundant species with the highest contribution in February (88.95%) and the only species collected in June. *Anopheles oswaldoi* s.l. and *Anopheles rangeli*, the latter absent in LUP, were collected only in February and April. In CAH, of the specimens that were identified to species, only *An. darlingi* was present.

**Table 2:** *Anopheles* species composition in three localities in the Iquitos area in 2011–2013

Locality	Collection month	*Anopheles* species HLC (total collected)	*Anopheles* species SHA (total collected)	Species composition (%)
LUP 2011	February	*An. darlingi* (327)	*An. darlingi* (17)	57.1
		*An. nuneztovari* s.l. (41)	*An. nuneztovari* s.l. (149)	31.6
		*An. oswaldoi* (2)	*An oswaldoi* (8)	1.6
		*An. triannulatus* (6)	*An. triannulatus* (12)	3
		*An. benarrochi* (1)	*An. benarrochi* (1)	0.3
		*Nyssorhynchus* (21)	*Nyssorhynchus* (8)	4.8
		*Anopheles spp.* (3)	*Anopheles spp.* (6)	1.6
	April	*An. darlingi* (2,381)	*An. darlingi* (430)	82.7
		*An. nuneztovari* s.l. (161)	*An. nuneztovari* s.l. (202)	10.7
		*An. oswaldoi* (22)	*An. oswaldoi* (42)	1.9
		*An. triannulatus* (3)	*An. triannulatus* (8)	0.3
		*An. benarrochi* (1)	*An. benarrochi* (1)	0.05
		*Nyssorhynchus* (85)	*Nyssorhynchus* (34)	3.5
		*Anopheles spp*. (22)	*Anopheles spp.* (6)	0.85
	June	*An. darlingi* (107)	*An. darlingi* (26)	96.4
		*An. oswaldoi* (1)		0.7
		*Anopheles* spp. (4)		2.9
	August	*An. darlingi* (30)	*An. darlingi* (3)	86.84
		*An. oswaldoi* (1)		2.6
		*Nyssorhynchus* (2)		7.9
			*Anopheles spp.* (1)	2.6
	October	*An. darlingi* (22)	*An. darlingi* (5)	61.3
		*An. nuneztovari* s.l. (5)	*An. nuneztovari* s.l. (1)	13.6
		*An. oswaldoi* (1)	*An. oswaldoi* (2)	6.8
		*Nyssorhynchus* (1)		2.2
		*Anopheles spp.* (5)	*Anopheles spp.* (2)	15.9
	December	*An. darlingi* (15)	*An. darlingi* (1)	33.3
		*An. nuneztovari* s.l. (4)	*An. nuneztovari* s.l. (1)	10.4
		*An. oswaldoi* (4)	*An. oswaldoi* (2)	12.5
		*Nyssorhynchus* (8)	*Nyssorhynchus* (3)	22.9
		*Anopheles spp.* (8)	*Anopheles spp.* (2)	20.8
2012	February	*An. darlingi* (379)	*An. darlingi* (4)	93.2
		*An. oswaldoi* (3)		0.7
		*Nyssorhynchus* (8)	*Nyssorhynchus* (1)	2.2
		*Anopheles spp.* (16)		3.9
	April	*An. darlingi* (1353)	*An. darlingi* (300)	99.9
		*Anopheles spp*. (1)		0.1
	June	*An. darlingi* (499)	*An. darlingi* (151)	96.6
		*An. oswaldoi* (1)	*An. oswaldoi* (3)	0.6
		*An. triannulatus* (1)	*Nyssorhynchus* (2)	0.2
		*Nyssorhynchus* (12)		2
		*Anopheles spp.* (4)		0.6
	August	*An. darlingi* (17)	*An. darlingi* (14)	100
	September	*An. darlingi* (13)	*An. darlingi* (3)	100
	November	*An. darlingi* (14)		100
VBP 2011	February	*An. darlingi* (1)	*An. darlingi* (7)	88.9
		*An. rangeli* (1)		11.1
	April	*An. darlingi* (588)	*An. darlingi* (81)	98.2
		*An. oswaldoi* (3)	*An. oswaldoi* (1)	0.57
		*Nyssorhynchus* (2)		0.3
		*Anopheles spp.* (1)	*Anopheles spp.* (5)	0.89
	June	*An. darlingi* (50)	*An. darlingi* (3)	100
	August	*An. darlingi* (41)	*An. darlingi* (7)	98
		*Nyssorhynchus* (1)		2
	October	*An. darlingi* (28)	*An. darlingi* (4)	97
		*Nyssorhynchus* (1)		3
	December	*An. darlingi* (16)	*An. darlingi* (4)	95.2
		*Nyssorhynchus* (1)		4.8
CAH 2012	May	*An. darlingi* (1653)	*An. darlingi* (543)	99.3
		*Nyssorhynchus* (16)	*Nyssorhynchus* (1)	0.7
	August	*An. darlingi* (16)	*An. darlingi* (1)	100
	October	*An. darlingi* (85)	–	100
	December	*An. darlingi* (120)	*An. darlingi* (12)	100

Data in this table reflects the density of anopheline species present collected during the study. Collections were performed 4 days every month reported in the table; 2 days were performed 12 h (18.00–6.00) and 2 days were collected for 4 h (18.00–22.00).

*Nyssorhynchus* denotes specimens that could not be identified.

*LUP* Lupuna, *VBP* Villa Buen Pastor, *CAH* Cahuide, *HLC* human landing catch, *SHA* Shannon trap.

Molecular identification was performed on a sub-sample of the specimens that could not be identified morphologically (Table 3). *Anopheles nuneztovari* s.l. has been identified from the Iquitos region previously [9]. However, because of the recognition of the Nuneztovari complex [36], which includes *An. dunhami*, *An. nuneztovaris.s*. and *Anopheles goeldii*, 22 samples were randomly tested using barcode *COI* sequences. All these samples were confirmed as *An. dunhami* using unique haplotypes with a bootstrap neighbour-joining Kimura 2-parameter (K2P) [37] distance model (1,000 replicates) and Bayesian phylogenetic tree analysis [38, 39] with published sequences [36, 40]. This confirmation of *An. dunhami* extends its distribution (previously known from Brazil and Colombia) into Amazonian Peru for the first time. In addition, the detection and confirmation of *An. oswaldoi* C in LUP and VBP is the first record of this species in the area. *Anopheles benarrochi* B, a member of the *An. benarrochi* complex, was first reported in Peru recently [8].

**Table 3:** Molecular identification of morphologically identified samples from LUP and VBP by different methods

Locality	*AluI* and *BsrBI* digest	Barcode *COI*	ITS2
LUP			
*An. nuneztovari* s.l.	252		
*An. dunhami*		22	
*An. benarrochi* B	3	3	2
*An. oswaldoi*	33	1	3
*An. oswaldoi* C		5	4
VBP			
*An. rangeli*	1		
*An. oswaldoi*	2		
*An. oswaldoi* C		2	

Only *An. darlingi* was identified in CAH.

**Figure 2:**
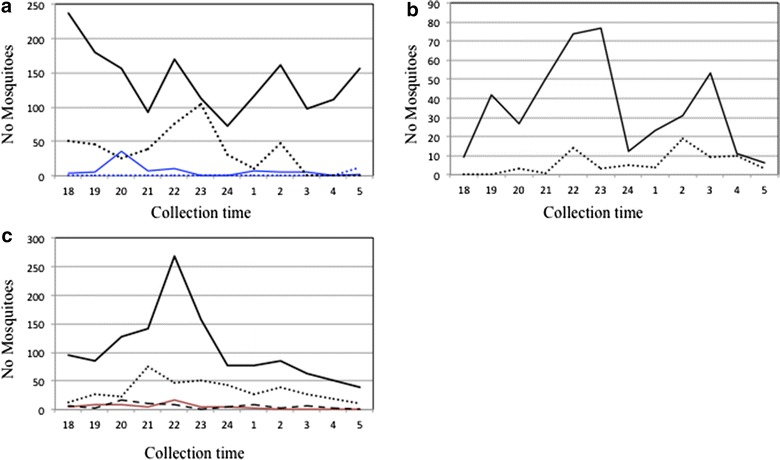
Mosquito collection by time and collection method in the three localities. **a** LUP: *black line*—HLC and *black dotted-line*—SHA, April 2011; *blue line* corresponds with HLC and *blue dashed-line* with SHA in February 2011. **b** VBP: *black line*—HLC; *black dotted-line*—SHA, April 2011. **c** CAH: *black line*—HLC and *black dashed-line*—SHA, May 2012. *Red line*: October 2012. *Black dashed line*: December 2012. Only months with positive *Plasmodium* mosquitoes are represented in each locality.

**Figure 3:**
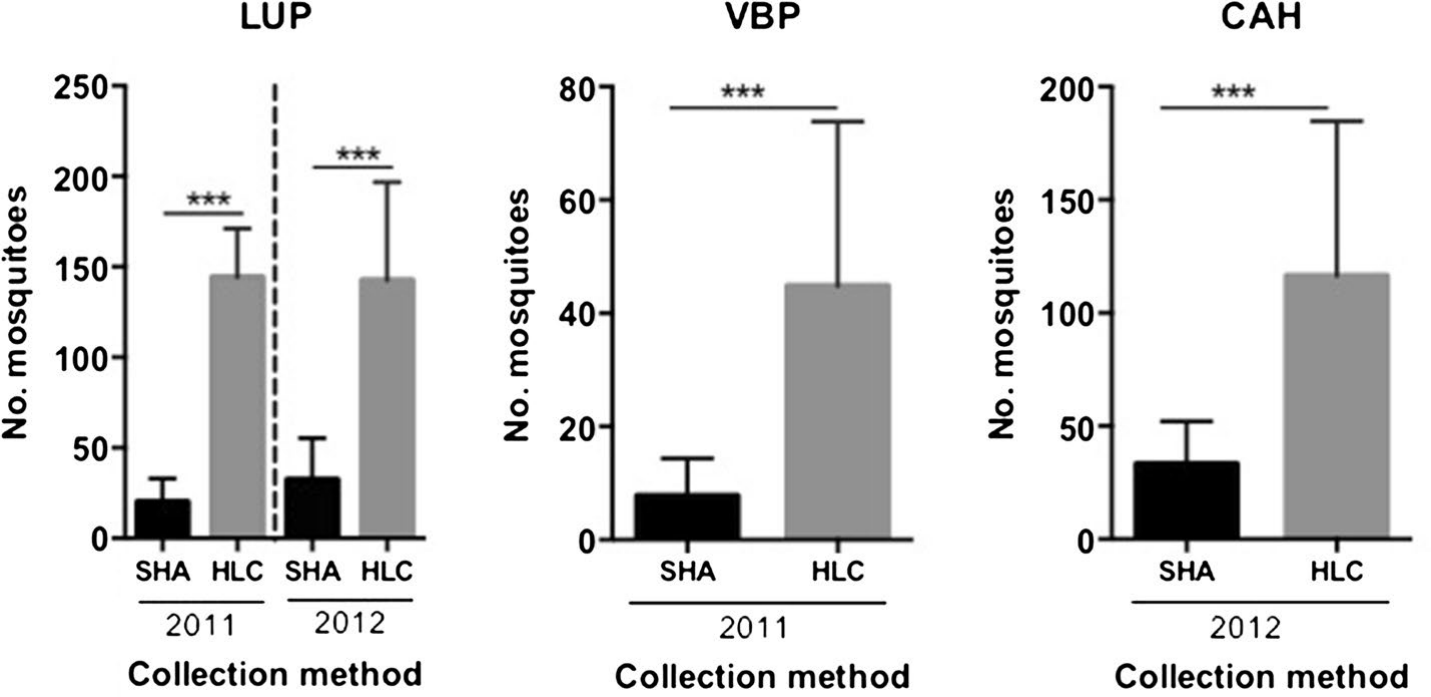
Mosquito captures by collection method and year of collections in the three study localities. Overall number of mosquitoes collected by HLC or SHA was statistically tested by non-parametric Wilcoxon-test; ***significant value *p* < 0.001.

Biting patterns were similar in LUP and VBP, regardless of collection method, in April 2011 (Figure 2). During the dry season, mosquito numbers were so low that comparisons of biting activity could not be done. In May 2012, for CAH, there was a marked peak at 22:00 with HLC, and 21:00 using SHA. Anopheline abundance using HLC and SHA in LUP and VBP (Figure 2) peaked sharply in April, and in May in CAH. Collection method and time was compared in each locality. Significance was detected in LUP only between 22:00 and 00:00 (*p* < 0.005). The same comparisons in VBP were only significant at 02:00 (*p* < 0.05). An independent analysis comparing dry versus rainy season could not be conducted due to low dry season specimen numbers. Overall, comparisons between collection methods in all localities were highly significant (Figure 3).

Only 11 *An. darlingi* and four *An. nuneztovari* s.l. were collected with CDC traps indoors in LUP in April, the month with the greatest abundance of mosquitoes. In VBP and CAH no mosquitoes were collected with these traps, probably because of the characteristics of the houses (open windows and a gap between wall and roof).

In LUP, between 18:00 and 22:00, 82% of the mosquitoes were collected outdoors by HLC. More than 75% of these mosquitoes were *An. darlingi*, followed by 7.8% *An. nunezovari* s.l., 2.2% *An. oswaldoi* s.l. and 4.5% *Nyssorhynchus.* All indoor specimens were *An. darlingi* except one *An. nuneztovari* s.l. In CAH, 93.5% were collected outdoors and all were identified as *An. darlingi*. Statistical analysis of indoor versus peridomestic HLC (18.00–22.00) collections could not be done because of low numbers of indoor specimens.

**Figure 4:**
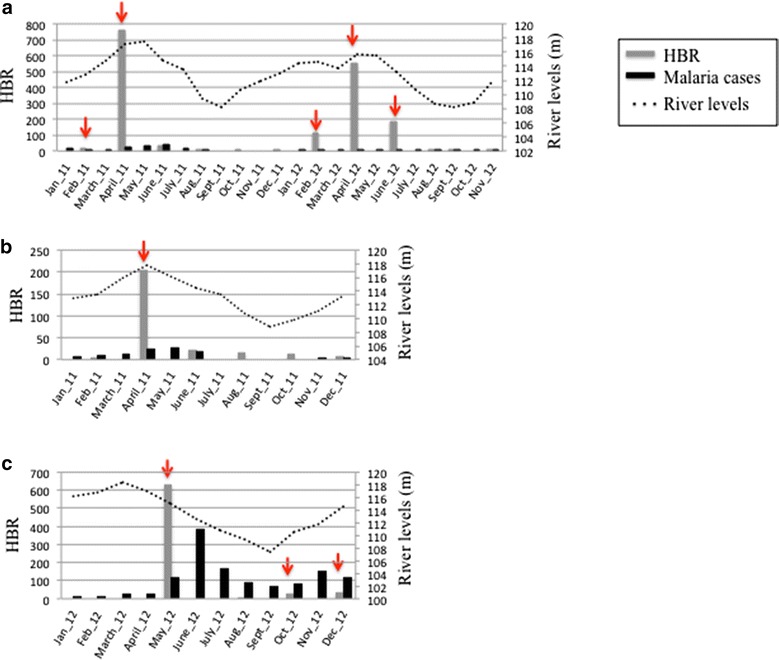
Human biting rate (bites/person/night), malaria cases and river levels (meters above sea level) in the three sites. Monthly Nanay River levels for **a** LUP (range 108.2–117.4 m) and **b** VBP; **c** CAH Itaya River levels (range 107.4–118.4 m). In both rivers, March–April were the maximum and September the minimum water levels. Malaria cases were diagnosed by microscopy and reported to the correspondent health post. *Arrows* represent *Plasmodium*-infected individual *An. darlingi.*

HBR varied dramatically, clearly correlated with seasonality, in all localities (Figure 4). In LUP the overall anopheline peak was in April (HBR = 831 bites/person/night), mostly contributed by *An. darlingi* (HBR = 757). In VBP, the highest HBR was also April (HBR = 205) for *An. darlingi*, with little variation when including the small number of other anopheline species. In CAH, the highest HBR for *An. darlingi* (HBR = 630.5) was in May (collections were not done in April), decreasing to HBR = 5 in August. Pearson’s correlation analysis between HBR and river levels confirmed the connection between these two parameters in LUP (*r* = 0.7182; *p* = 0.0085) but not in CAH (*r* = 0.6214; *p* = 0.3786) or in VBP (*r* = 0.7827; *p* = 0.0657). The latter was likely non-significant due to the low number of observations (only from 2011), in contrast to two consecutive years of observations for LUP.

A total of 455 and 169 anopheline pools from LUP and VBP respectively, were tested for *Plasmodium* using *Cyt*-*b* and 25 of these (all *An. darlingi*) were positive. In LUP, 13/2,275 (0.57%) *An. darlingi* were infected with *P. vivax*; two were mixed infections with *P. falciparum*. In VBP, 12/845 (1.42%) *An. darlingi* were infected with *P. vivax*, and four were mixed infections. Only two of the infected mosquitoes were collected by SHA, one in each of LUP and VBP. Infected mosquitoes were identified in the months of February and April in LUP in 2011, and February, April and June in 2012; in VBP, infective mosquitoes were detected only in April (Figure 4). The distribution of the infected *An. darlingi* differed depending on locality and trap collection. Infected mosquitoes were detected from 18:00 to 03:00 in LUP and between 19:00 and 23:00 in VBP. None of the mosquitoes collected indoors (only from LUP and CAH) were infected (103 and 118 tested, respectively).

All samples from 2012 were tested by ELISA. These data showed that 9/558 (0.32% IR) pools were positive in LUP and 18/496 (0.72% IR) in CAH (all *An. darlingi*). In LUP, *P. vivax* VK247 was the most frequent variant followed by *P. vivax* VK210 and only one mosquito was infected with *P. falciparum*. In CAH, *P. vivax* VK210 was detected in 12 mosquitoes, *P. vivax* VK247 in seven and *P. falciparum* in three. The distribution of infected mosquitoes through the 12-h collections varied by year and locality (Figure 4). In 2012 infected mosquitoes were detected earlier in the night than in 2011. All other *Anopheles* species were tested and were negative for *Plasmodium*. Therefore, EIR calculated only for *An. darlingi,* in LUP (2011) ranged from 0.04 in February to 0.80 in April. In VBP, EIR for April was 0.86 (Table 4). In 2012 in LUP EIR was higher than the previous year, i.e., 0.31 in February, 1.98 in April and 0.59 in June. In CAH, EIR was 2.52 in May, 0.33 and 0.25 in October and December, respectively (Table 4).

**Table 4:** *Anopheles darlingi human* biting rate and entomological inoculation rate by month in the three localities studied

Collection date	LUP			VBP			CAH		
	IR 2011/2012	HBR (±SE) 2011/2012	EIR 2011/2012	IR 2011	HBR (±SE) 2011	EIR 2011	IR 2012	HBR (±SE) 2012	EIR 2012
January	–	–	–	–	–	–	–	–	–
February	5.88/0.44	17 (±0.5)/125.5 (± 9.5)	0.04/0.31	0	3.5 (±0.5)	0	–	–	–
March	–	–	–	–	–	–	–	–	–
April	0.66/0.54	757 (±83)/550.5 (±11.5)	0.807/1.98	1.4	205 (±31)	0.86	–	–	–
May	–	–	–	–	–	–	1.26	630.5 (±201.5)	2.52
June	0/0.53	31.5 (±21.5)/188.5 (±126.5)	0/0.59	0	22 (±12)	0	–	–	–
July	–	–	–	–	–	–	–	–	–
August	0	9.5 (±0.5)/4 (±2)	0/0	0	15.5 (±3.5)	0	0	5 (±2)	0
September		–/5.5(±1.5)	–/0						
October	0	7.5 (±2.5)/–	0/–	0	11.5 (±7)	0	1.47	28.5 (±3.5)	0.33
November		–/3.5 (±0.5)	–/0						
December	0	4(±1)/–	0/–	0	7.5 (±0.5)	0	1.75	34 (±4)	0.25

HBR: average bites per person per night (b/p/n) obtained from a mean of two collectors between 2 days/12 h per day per collection month. Calculations were made only with mosquitoes collected for 12 h by HLC.

*IR* infection rate, *EIR* entomological inoculation rate.

– Data not available.

## Discussion

EIR results demonstrated sustained seasonal malaria transmission among sites, ranging from 2.52 during the highest transmission season of April to 0 in the dry season. This rate is comparable to recent values for *An. darlingi* in Amazonian Peru, Colombia and Venezuela [4, 43, 44]. The temporal anopheline density peak described in this study coincides with high river levels as previously reported [9]. The highest HBR was 757 bites/person/night in LUP in April 2011 and 630.5 in CAH in May 2012. These values rank among the highest ever recorded for *An. darlingi* [12], for example, 837.7 in Matapi River (Amapa State, Brazil) [41], and 257.7 in Upper-Maroni (French Guiana) [42]. Because HBR is a major component of the EIR, such levels of HBR can be one indicator of the risk of contracting malaria, even when infection rate (IR) is low [41]. The IR levels detected in this study for *An. darlingi* (Table 4) were comparable to others in the region, for example 0.1–3.1% in 1996–97, during a malaria outbreak [9] and a total of 1.4% among multiple sites along the Mazan River [4]. The highest IR in the present study was from LUP in February 2011 (5.88), however the number of mosquitoes was low (Table 2). Nevertheless, this IR was almost double the highest level reported by Parker and collaborators (2.88, Jan/Feb 2009). The high IR in CAH during the months of October and December (Table 4) coincides with an unexpected increase in reported local malaria cases (DIRESA, Peru Ministry of Health).

The lack of infected indoor *An. darlingi* captures suggests that most, if not all, malaria transmission in the study sites occurs outdoors (although there were few mosquitoes to test). This pattern of transmission possibly represents a shift in *An. darlingi* biting behaviour related to the use of LLINs. Data from 1996–97 detected nearly a 1:1 proportion of *An. darlingi* indoor:outdoor from three localities south of Iquitos [9], prior to any distribution of LLINs [27]. Although this study was not designed to test the hypothesis of shifting behaviour of *An. darlingi* driven by LLINs, there are recent examples of such behaviour in Senegal for *Anopheles funestus* [45] and in the Solomon Islands for *Anopheles farauti* [46]. The results presented in this manuscript may presage a new trend in *An. darlingi*, and additional investigation should be performed.

The dominance of *An. darlingi* compared to all other species in this study is evidence of its successful spread in both rural and urban areas surrounding Iquitos, with the apparent reduction of other anopheline species such as *An. benarrochi* s.l. and *An. oswaldoi* s.l. [9, 47]. Extension of activity of *An. darlingi* throughout the night, as demonstrated in this report, is related to high mosquito densities and has also been reported in Amazonian Brazil [48].

The molecular identification of *An. dunhami* suggests that previous studies that have identified *An. nuneztovaris.s.* in Loreto could be erroneous, since currently available morphological keys cannot distinguish between adult females of *An. dunhami* and *An. nuneztovari* [36]. Junin is the second most important department reporting malaria cases in Peru (2,038 in 2014). *Anopheles trinkae*, identified as the main malaria vector there [49], may be *An. dunhami* [40]. If so, it would be important to determine the distribution and bionomics of *An. dunhami* in Peru, and to update current dichotomous keys to reflect its presence.

This study clearly showed that overall, in the Iquitos area, the HLC method caught an average of 35 times as many mosquitoes as the SHA, and CDC-LTs were not effective. Findings of significantly more infected mosquitoes collected with HLC compared with SHA agree with a recent review of anopheline collection methods conducted in Brazil [50], that determined that HLC is still the most efficient collection method. In the Neotropics few alternatives to HLC have been evaluated. Hiwat et al. [51] in Suriname compared HLC, CDC-LT, Mosquito Magnet Liberty Plus mosquito traps and BG sentinel traps baited with CO_2_ to collect *An. darlingi*; HLC attracted significantly greater numbers of the target species. Additionally, HLC compared to Magnet Traps in Venezuela attracted significantly more *An. darlingi* [52]. In Brazil, the BG-Malaria trap [53] was as effective as HLC with respect to number and parity of *An. darlingi* collected, and thus appears to be a promising alternative for collecting and monitoring this important vector. A new collection method, barrier screens, was highly successful in collecting blood-fed anopheline vectors in the South Pacific [54]. Future research activities in the peri-Iquitos region will test the efficacy of this method and expand its potential utility for calculating vector biology metrics, if warranted.

## Conclusions

This study clearly demonstrated microgeographic differences in *An. darlingi* peak biting times, biting patterns, infectivity, and EIR. Study sites presented moderate EIR at least once annually, coincident with the highest mosquito abundance, similar to EIR reported in hyper-endemic transmission settings in the Amazon. *Anopheles darlingi* was the most abundant species and the only one infected with *Plasmodium*, confirming its importance as the major malaria vector in the area. HLC is still the most effective method for *An. darlingi* collection in this region.
